# Impact of Surgery on Long-Term Results of Hearing in Neurofibromatosis Type-2 Associated Vestibular Schwannomas

**DOI:** 10.3390/cancers11091376

**Published:** 2019-09-16

**Authors:** Isabel Gugel, Florian Grimm, Marina Liebsch, Julian Zipfel, Christian Teuber, Lan Kluwe, Victor-Felix Mautner, Marcos Tatagiba, Martin Ulrich Schuhmann

**Affiliations:** 1 Department of Neurosurgery, University Hospital Tübingen, 72076 Tübingen, BW, Germany; florian.grimm@med.uni-tuebingen.de (F.G.); marina.liebsch@med.uni-tuebingen.de (M.L.); julian.zipfel@med.uni-tuebingen.de (J.Z.); teuber_christian@web.de (C.T.); marcos.tatagiba@med.uni-tuebingen.de (M.T.); martin.schuhmann@med.uni-tuebingen.de (M.U.S.); 2 Centre of Neurofibromatosis and Rare Diseases, University Hospital Tübingen, 72076 Tübingen, BW, Germany; v.mautner@uke.de; 3 Division of Pediatric Neurosurgery, University Hospital Tübingen, 72076 Tübingen, BW, Germany; 4 Department of Neurology, University Medical Center Hamburg-Eppendorf, 20251 Hamburg, HH, Germany; kluwe@uke.de; 5 Department of Maxillofacial Surgery, University Medical Center Hamburg-Eppendorf, 20251 Hamburg, HH, Germany

**Keywords:** brainstem auditory evoked potentials, facial nerve, hearing preservation, neurofibromatosis type 2, surgery, vestibular schwannoma

## Abstract

Hearing preservation is a major goal in the treatment of neurofibromatosis type 2 (NF2) associated vestibular schwannoma (VS), particularly in children and adolescents. In this study, we retrospectively reviewed hearing and volumetry data sets of 39 operated tumors (ears) in 23 patients under the age of 25 and in a follow-up period of 21 to 167 months. Hearing data over a compatible period on 20 other tumors, which did not receive surgery due to their less aggressive nature, were included for comparison. Surgery was carried out via a retrosigmoid approach with the brainstem auditory evoked potential (BAEP) guide. Immediately after surgery, functional hearing was maintained in 82% of ears. Average hearing scores were better in the non-surgery ears. However, the hearing scores in both groups worsened gradually with a similar dynamic during the 42-month postoperative follow-up period. No accelerated impairment of hearing was evident for the operated cases. Rather, the gap between the two hearing deterioration lines tended to close at the end of the follow-up period. Our result suggested that the BAEP-guided surgery did not cause additional hearing deterioration in the long-term and seemed to slow down hearing deterioration of those tumors that were initially more aggressive.

## 1. Introduction

Neurofibromatosis Type 2 (NF2) is a rare autosomal-dominant tumor predisposition syndrome, with a prevalence of 1:56,000, an incidence of 1 case in 33,000 to 40,000 live births per year [1,2]. The disease is characterized by bilateral vestibular schwannomas (VS), other benign central or peripheral nervous system tumors (e.g., meningiomas, non-VS schwannomas, and spinal ependymomas) and ocular, bone, and cutaneous manifestations [3,4,5,6]. The genetic cause for NF2-associated tumors is the biallelic inactivation of the *NF2* tumor-suppressor gene on chromosome 22q12 [7,8]. Genotype–phenotype correlations exist and are well-investigated [9,10].

Due to the bilateral nature of VS in NF2 patients, hearing preservation is extremely important to prevent total deafness, especially for young patients. For hearing preservation, several strategies have been proven to be effective for NF2-associated VS—(1) partial resection mainly for decompression of the internal auditory canal and (2) electrophysiological guidance with brainstem auditory evoked potential (BAEP) and motor evoked potentials (MEP) of the facial nerve, by experienced interpreters [11,12,13]. Nevertheless, surgery is still associated with a risk of damaging hearing and facial functions. Consequently, a conservative option of “wait and see” is often considered, especially for small and less aggressive tumors [14]. However, most patients need surgery, sooner or later, since progressive tumors cause hearing impairment, brainstem compression and other manifestations. In addition, early surgery for small tumors has improved the chance of hearing preservation. Therefore, the choice of treatment and timing always present a dilemma and have to be considered individually for balancing the risks, benefits, and patient wish. For such considerations and therapy decision, data regarding post-operative hearing function in the long-term would provide valuable information.

The aim of this study is to investigate the hearing function before surgery, immediately after surgery, and in the years following surgery, in young NF2 patients under 25 years of age, during diagnosis. Furthermore, confounding factors such as tumor volume and mutations are also investigated.

## 2. Results

### 2.1. Patients, Tumors, and Operation

Detailed clinical and demographic data are provided in Table S1 and are summarized in Table 1 and Table 2.

Partial resection was achieved in a majority of the tumors—32/39; 82%. We did not record any major surgical complications in the short- and long-term follow-up (e.g., intra-operative bleeding, intra-operative air embolism, cerebrospinal fluid fistula, infection, and neurologic deficit) other than deterioration of hearing or facial nerve function. Minor complications directly after surgery occurred in 8 cases, such as wound healing disorders (2/8) and severe neck pain/headache (6/8) and could be successfully treated conservatively.

### 2.2. Post-Operative Facial Function

Facial nerves function was preserved in 37/39 (95%) tumors. In two cases, an improvement from the House–Brackmann Classification System (H–B) grade II to I was achieved by surgery (Table S1). 

### 2.3. Postoperative Deafness

Seven resections resulted in postoperative deafness (Table S1). However, 2 ears already had non-functional hearing before surgery. Though the other 5 ears had remaining hearing before surgery, BAEP were already severely affected. Preoperative tumor volumes and resection amounts in these 5 cases were significantly larger than those in the other 32 cases with preserved hearing in 3.7 ± 3.8 cm^3^ versus 1.6 ± 2.0 cm^3^ (*p* = 0.027) and 66% ± 38% versus 38% ± 32% (*p* = 0.024), respectively.

### 2.4. Postoperative Hearing

For 32 (82%) ears, functional hearing could be preserved after surgery. Slightly significant reduction in pure-tone average (PTA) and in the speech discrimination score (SDS) was measured, which, however, were functionally not relevant (Table 1). One ear improved in SDS though not in PTA.

Preservation of the preoperative hearing grade could be achieved in 29 (74%), 28 (72%), and 24 (62%) ears, with regards to the Gardner and Robertson Scale (G–R), American Association of Otolaryngology–Head and Neck Surgery (AAO–HNS) and the BAEP classification, respectively. Three (8%) ears improved in BAEP. A better preoperative G–R grade correlated with a higher preservation rate in the same grades—81%, 50%, and 33% for grade I, II, and III, respectively.

Three bony decompression surgeries without tumor resection resulted in hearing preservation. One subtotal resection led to a BAEP drop from 2 to 5. All other subtotal and near total resections were in ears with poor preoperative BAEP scores of 4 and 5. Interestingly, the only total resection (for a small tumor in T1 of the Hannover classification) resulted in a hearing preservation with a BAEP remaining in score 1. The majority (32) of resections were partial with various outcomes of hearing (Table S1).

Correlations of the additional parameters with postoperative hearing are summarized in Table 3.

### 2.5. Postoperative Hearing in Long-Term

**Figure 1:**
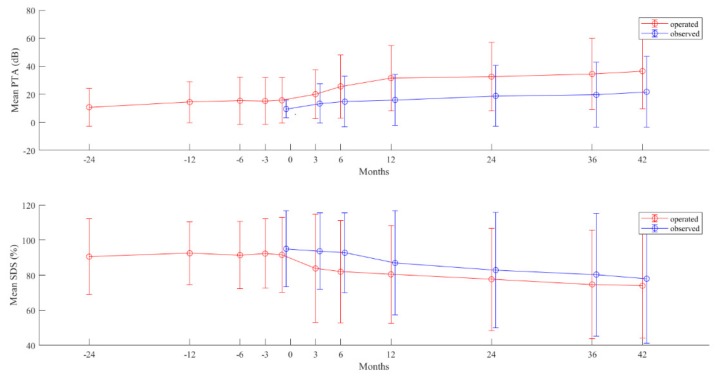
Time course of hearing deterioration in the 39 operated (**red line**) and the 20 non-operated (**blue line**) vestibular schwannoma (VS) in NF2 patients. Pure-tone average (**upper figure**) and speech discrimination score (**lower figure**). Values are in mean (**points**) and standard deviation (**bars**) of tumors in each group at each measuring time point. Time point 0—time point of surgery and starting point of observation in the non-operated (observed) group.

Hearing parameters PTA and SDS of the 39 operated ears were postoperatively followed up to 42 months. In comparison, 20 age-matched cases (detailed demographic data are given in Table 4) that did not receive surgery were also followed. Initial hearing of the non-operated control group was better because good hearing was also the reason for not receiving surgery. However, the mean values of hearing scores in both groups worsened gradually (*p* < 0.05) in a similar dynamic over the 42-month postoperative follow-up period. No accelerated hearing deterioration was evident for the operated cases, despite a worse hearing starting point; instead, the gap between the decreasing lines in the two groups tended to close at the end of the follow-up period (Figure 1).

## 3. Discussion

We report a high postoperative functional hearing preservation rate of 82% after the BAEP guided surgery. Additionally, our rate of facial function preservation of 95% was high in comparison to the reported ones, which varied from 76% to 100% [11,13,16,17,18,19,20].

The major finding of the present study was that long-term (mean 6.28 years) hearing deterioration became similar in cases with and without surgery. The variation of hearing deterioration was large but equal in both groups of tumors, regardless of surgery. This observation suggests that the surgery of VS did not accelerate hearing loss but rather modified the initial, more rapid hearing deterioration (caused by the aggressively growing VS) toward a slower hearing deterioration, which was similar to that of the non-operated less aggressive VS. 

Hearing preservation is more likely to be achieved in patients that had a good hearing before the surgery [11,16,21]. Our results including the BAEP data further confirmed this rule and suggests that intervention should be considered when BAEP starts to deteriorate, before a drop in PTA or SDS can be detected. 

Due to the high risk of bilateral deafness over the time, hearing preserving surgery has a much higher value for NF2 patients than for patients with unilateral sporadic VS. Consequently, total resection should not be the intended goal, as such a surgery has a much lower hearing preservation rate of 30%, compared to the 73% achieved by subtotal resection [11]. Additionally, in this series, the resection amount significantly correlates with deterioration in postoperative hearing. 

Thus, our study confirmed again, and this is of major importance, that the surgeon needs to be conscious with regard to the intraoperative BAEP behavior and needs to show a defensive surgical behavior to preserve functional hearing. This results in smaller resection amounts in the majority of cases. A similar experience of better hearing preservation by deliberate partial resection has been reported by Wigand et al. [22], using a middle fossa approach. Thus, we conclude that the influence of the chosen surgical approach (retrosigmoid or middle fossa) is secondary to the intraoperative strategy and does not influence the functional outcome. Both approaches have their benefits and drawbacks. We prefer the retrosigmoid approach since it can be used in small intracanalicular tumors, as larger tumors fill the cerebellopontine angle cistern. Furthermore, the risk for a facial nerve dysfunction is lower in our experience, as the facial nerve is rarely encountered in a strictly hearing preserving strategy and, thereby, is exposed at a later stage of surgery. However, this timepoint is often not reached due to the mostly earlier occurring BAEP deterioration which results in a termination of surgery. Due to its anatomical localization, the facial nerve seems to be more endangered in a middle fossa approach.

A potential disadvantage of the retrosigmoid approach is the high rate of longer lasting neck pain or occipital headache (still existing at the time of discharge) from damage and irritation to the oblique neck muscles [23]. However, in the longer follow-up, in all six patients these complaints subsided, similar to what is described elsewhere in the literature [23].

In summary, results of the present study suggest that early surgery of VS in NF2 patients, which is strictly oriented at functional hearing preservation and is not aimed at tumor removal, has a long-term beneficial effect of slowing down the dynamics of hearing impairment. 

In our overall management strategy, this type of surgery is, therefore, often chosen as the first step, followed by a bevacizumab treatment at a later delayed timepoint, for surgery, when hearing deteriorates again. Thus, the side effects of this treatment can be postponed by several years and its costs are avoided.

## 4. Material and Methods

### 4.1. Patients and Clinical Characteristics

A total of 23 NF2 patients under 25 years during diagnosis were included in this retrospective analysis. Operations were performed on 39 tumors between 2004 and 2018, at the Department of Neurosurgery and Centre of Neurofibromatosis in Tübingen.

A total of 20 control ears in 15 NF2 patients with the same cut-off age at time of diagnosis were chosen as a control group. These patients had stable hearing and tumor growth and, therefore, had no indication for a surgical intervention during the observation period. Neither the operated nor the non-operated patients were treated with chemotherapy during the time intervals under investigation. 

The Ethics Board of the Medical Faculty and the University Hospital of Tübingen approved this retrospective analysis (No 018/2019BO2). All operations were performed at our institution via the retrosigmoid approach, by decompression of the internal auditory canal (IAC) with various resection amounts, under continuous neurophysiological monitoring by two experienced neurosurgeons (MT, MUS). 

Indications for surgery were (a) large tumors (T4 Hannover Classification [24]) on both sides with brainstem compression or (b) continuing tumor growth and deterioration of auditory evoked potential or impairment of pure-tone average (PTA) or speech discrimination score (SDS), during observation. 

The main influencing factors evaluated for impact of surgery on hearing included, (1) age at diagnosis, (2) preoperative tumor growth rate, (3) preoperative tumor volume, (4) resection volume, and (5) type of constitutional mutation. The data were obtained from clinical reports and MRI. Tumor volumetry, growth rate, and resection amount were performed, classified, and calculated in 579 data sets, as previously described [25]. For 21 out of 23 patients, mutation analysis was carried out as previously described [26]. The remaining two patients or their parents rejected mutation analysis. 

### 4.2. Hearing Evaluation

Patients with hearing aids or implants (e.g., cochlear or auditory brainstem implant) were excluded. Hearing was assessed in all patients by regular determination of 4-frequency PTA, SDS, and BAEP, within 4 weeks before surgery and then every three-to-six months after surgery. Hearing data were classified using the Gardner and Robertson Scale (G–R) [15], American Association of Otolaryngology–Head and Neck Surgery (AAO–HNS) and BAEP Classification System [12], direct and mean values of PTA, SDS, and BAEP waves, including interpeak latencies (IPL). Preservation of hearing was defined as PTA and SDS change of ≤15 dB or ≤15%, compared to the preoperative value [17] and by preservation of G–R, AAO–HNS and BAEP class. Facial nerve function was categorized by the House–Brackmann Classification System (H–B) [27] before surgery and three months after surgery. The mean follow-up after surgery was 75 months (range 21 to 167 months).

### 4.3. Statistical Analysis

Hearing before and after surgery was compared using paired by *t*-test with a two-tailed hypothesis. Pearson’s correlation coefficient was used to analyze the relationship between hearing values/classifications and the VS growth rate, volume, and resection amount. After normalization of the pre- and postoperative hearing data, an analysis of variance (ANOVA) with the Games–Howell post-hoc test was carried out with PTA and SDS values with a comparison of the age of patients. To investigate the long-term course of PTA and SDS values before (until 24 months) and after (until 42 months) surgery, an analysis of covariance (ANCOVA) was performed and data were compared to the 20 control ears. 

## 5. Conclusions

BAEP-guided partial resection via the retrosigmoid approach for the VSs enabled a high rate of functional hearing preservation (74–82%). In the long term, such surgery does not negatively impact hearing and, therefore, provides a preferred primary treatment option for NF2-associated VS.

## Figures and Tables

**Table 1:** Demographic data of 23 young, operated-on neurofibromatosis type 2 (NF2) patients (39 tumors).

Sex	
male	8
female	15
Operation side	
left	19
right	20
Family history for NF2	
yes	3
no	20
Detected mutation types in 21 patients	
splicing mutations	6
nonsense mutations	5
frameshifting mutations	4
large genome alteration	1
Age at diagnosis in year (mean ± SD, range)	12 ± 7, 1–22
Age at first surgery in year (mean ± SD, range)	16 ± 5, 8–26
Age at second surgery in year (mean ± SD, range)	16 ± 4, 11–24
Follow-up period in months (mean ± SD, range)	75 ± 6, 21–167
Tumor volume in cm^3^ (mean ± SD, range)	
preoperative	2 ± 2.6, 0.1–10.5
postoperative	1 ± 1.6, 0–18.6
significance in difference	*p* = 0.002
Growth rate in cm^3^/year (mean ± SD, range)	
preoperative	0.6 ± 0.7, 0.03–3.4
postoperative	0.3 ± 0.4, −0.01–2.2
significance in difference	*p* = 0.03
Resection amount	
only bony decompression	2
decompression with laser coagulation	1
partial	32
subtotal	2
near total	1
total	1
PTA in dB (mean ± SD, range)	
preoperative	17 ± 16, 1.3–80
postoperative	21 ± 18, 1.3–78
significance in difference	*p* = 0.009
SDS in % (mean ± SD, range)	
preoperative	85 ± 27, 0–100
postoperative	81 ± 32, 0–100
significance in difference	*p* = 0.043

PTA—pure-tone average; SDS—speech discrimination score; SD—standard deviation.

**Table 2:** Preoperative and postoperative hearing classifications in 39 operated ears.

**G–R Scale [15]**	Postoperation Class (No)				
Preoperation Class (No)	I (26)	II (3)	III (3)	IV (0)	V (7)
I (32) II (4) III (3) IV (0) V (0)	26 0 0 0 0	1 2 0 0 0	1 1 1 0 0	0 0 0 0 0	4 1 2 0 0
**AAO–HNS Classification**	Postoperation Class (No)				
Preoperation Class (No)	A (26)	B (2)	C (0)	D (11)	
A (32) B (3) C (0) D (4)	26 0 0 0	1 1 0 0	0 0 0 0	5 2 0 4	
**BAEP Classification System [12]**	Postoperation Class (No)				
Preoperation Class (No)	I (5)	II (23)	III (4)	IV (0)	V (7)
I (11) II (23) III (1) IV (2) V (2)	3 2 0 0 0	5 18 0 0 0	1 1 1 1 0	0 0 0 0 0	2 2 0 1 2

G–R—Gardner and Robertson Scale [15]; AAO-HNS Classification—American Association of Otolaryngology–Head and Neck Surgery; BAEP—brainstem auditory evoked potentials Classification System according to Samii and Matthies et al. [12].

**Table 3:** Correlations of parameters/features with postoperative hearing.

**Positive Correlation**	
Preoperative BAEP correlated positively with Postoperative PTA	r = 0.3, *p* = 0.04
Preoperative PTA correlated positively with Postoperative PTA	*p <* 0.001
Preoperative SDS correlated positively with Postoperative SDS	*p <* 0.001
**Negative (Inversed) Correlation**	
Truncation *NF2* mutations correlate with worse PTA (compared to the splicing mutation)	*p* = 0.012
Truncation *NF2* mutations correlate with worse SDS (compared to the splicing mutation)	*p* = 0.008
Larger preoperative tumor volume correlates with worse postoperative PTA	r = 0.3, *p* = 0.04
Larger resection amount correlate with worse postoperative PTA	r = 0.354, *p* = 0.031
Larger resection amount correlate with worse postoperative SDS	r = −0.386, *p* = 0.018

**Table 4:** Demographic data of 15 young non-operated NF2 patients (20 tumors).

Sex	
male	4
female	11
Family history for NF2	
yes	4
no	11
Detected mutation types in 8 patients	
splicing mutations	3
nonsense mutations	2
frameshifting mutations	3
Mosaic (No)	3
No mutation detected (No)	1
No genetic analysis performed (No)	3
Age at diagnosis in year (mean ± SD, range)	10 ± 7, 0−21
Follow-up period in months (mean ± SD, range)	60 ± 33, 14−117

## Supplementary Materials

The following are available online at https://www.mdpi.com/2072-6694/11/9/1376/s1, Table S1: Parameters for the 39 operated VS and associated hearing from 23 young NF2 patients.
