# Supplementary material for: Impact of Surgery on Long-Term Results of Hearing in Neurofibromatosis Type-2 Associated Vestibular Schwannomas
Source: Cancers (Basel). 2019 Sep 16;11(9):1376. doi: 10.3390/cancers11091376 (PMC6770212; doi:10.3390/cancers11091376)
Supplement: Supplementary file 1 [file cancers-11-01376-s001.pdf]

Impact of Surgery on Long-Term Results of Hearing in Neurofibromatosis Type-2 Associated Vestibular Schwannomas

Isabel Gugel, Florian Grimm, Marina Liebsch, Julian Zipfel, Christian Teuber, Lan Kluwe, Victor-Felix Mautner, Marcos Tatagiba and Martin Ulrich Schuhmann

Table S1. Parameters for the 39 operated VS and associated hearing from 23 young NF2 patients.

Tumor/ Patient ID	*Sex*	*Age at time of surgery (yr)*	**Resection amount *3**	*Preoperative*					*Postoperative *2*			
				Hannover-Classification [1]	*PTA (dB)*	*SDS (%)*	*H-B* [2]	*BAEP Score* [3]	*PTA (dB)*	*SDS (%)*	*H-B* [2]	*BAEP Score* [3]
**1.1**	m	12	3d	T2	10	100	2	1	35	82	4	2
**1.2**		8	1	T1	28.75	100	1	1	28	97	1	2
**2.1**	f	12	3c	T1	5	100	1	2	5	100	1	2
**2.2**		16	3a	T2	5	100	1	1	6.25	95	1	2
**3.1**	f	11, 15 *1	3e, 3a	T3a	15	70	1	2	43.75	65	1	2
**3.2**		13	2	T1	18.25	97	1	2	16.25	98	1	1
**4.1**	m	12	3d	T1	10	100	2	1	10	100	1	2
**4.2**		15	3e	T3a	10.50	100	1	1	deaf	deaf	1	5
**5.1**	m	10	3b	T1	11.25	72	1	1	15	30	1	3
**5.2**		13	3a	T2	41.45	45	1	2	48.75	20	1	2
**7.1**	f	10	3c	T1	5	100	1	2	10	100	1	2
**7.2**		11	3b	T1	11.25	98	2	2	12.70	96	1	2
**8.1**	f	13	3a	T1	12.25	85	1	5	deaf	deaf	1	5
**8.2**		13	3b	T1	5	100	1	3	6.5	100	1	3
**9.1**	f	11	3c	T1	14.70	100	1	1	17.50	97	1	1
**9.2**		12	6	T1	80	0	1	1	77.5	0	1	1
**10.1**	f	22	3e	T3b	5.5	95	1	2	23	81	1	1
**10.2**		23	3e	T3a	43	73	1	1	45	0	1	2
**11.2**	f	18, 22 *1	3c, 5	T4a	10.75	95	1	2	47.5	95	1	2
**12.1**	f	24	3d	T4a	12.5	99	1	2	26.25	100	1	2
**12.2**		23	3c	T3b	15	100	1	2	24.60	80	1	2
**14.2**	m	15	3d	T2	28.75	100	1	1	deaf	deaf	1	5
**15.2**	f	12	3c	T1	6.25	98	1	1	4	100	1	1
**19.1**	f	17	4	T4a	53.75	0	1	2	deaf	deaf	1	5
**21.1**	m	13	3c	T1	12.5	100	1	2	10	90	1	2

**21.2**		12	3d	T3a	6.25	95	1	2	6.25	100	1	2
**23.1**	f	15	3c	T3a	6.5	90	1	2	7.5	100	1	2
**23.2**		16	3c	T2	5	100	1	2	17	100	1	2
**24.1**	m	14	3b	T2	16.25	99	1	2	23.75	100	1	2
**24.2**		13	3b	T2	14.50	100	1	2	6.25	100	1	3
**25.1**	m	16	3a	T2	5	100	1	2	1.28	100	1	2
**25.2**		16	1	T2	1.25	100	1	2	3.25	100	1	2
**26.2**	f	26	3c	T3a	8.75	100	1	2	8	100	1	2
**28.1**	f	23	3b	T3a	48.75	58	1	2	47.3	0	1	2
**28.2**		24	3a	T4a	12.5	100	1	2	18.75	90	4	2
**35.1**	f	16	3a	T3a	25	91	1	2	deaf	deaf	1	5
**36.1**	m	22	5	T4a	40	75	1	4	deaf	deaf	1	5
**39.1**	f	23	4	T3a	85	0	2	5	deaf	deaf	2	5
**39.2**		23	3e	T2	35	30	2	4	30	85	2	3

Abbreviations: PTA: pure-tone average; SDS: speech discrimination score; H-B: House and Brackman Grading System [2]; BAEP: brainstem auditory evoked potentials Classification System according to Samii and Matthies et al. [3]; 1 = left-sided tumor; 2 = right-sided tumor. *[1] Tumor 3.1 and 11.2 were operated twice. Data before and after the first surgery was used. *[2] Postop values are evaluated 3 months after surgery. *[3] Resection amount as previously described in Gugel et al. [4].

References

1. Samii, M.; Matthies, C. Management of 1000 vestibular schwannomas (acoustic neuromas): Hearing function in 1000 tumor resections. *Neurosurgery* **1997**, *40*, 248–260; discussion 260-242.
2. House, J.W.; Brackmann, D.E. Facial nerve grading system. *Otolaryngol Head Neck Surg.* **1985**, *93*, 146–147.
3. Matthies, C.; Samii, M. Management of vestibular schwannomas (acoustic neuromas): The value of neurophysiology for evaluation and prediction of auditory function in 420 cases. *Neurosurgery* **1997**, *40*, 919–929; discussion 929–930.
4. Gugel, I.; Grimm, F.; Teuber, C.; Kluwe, L.; Mautner, V.F.; Tatagiba, M.; Schuhmann, M.U. Management of NF2-associated vestibular schwannomas in children and young adults: Influence of surgery and clinical factors on tumor volume and growth rate. *J. Neurosurg. Pediatr.* **2019**, 1–9, doi:10.3171/2019.6.PEDS1947.

© 2019 by the authors. Licensee MDPI, Basel, Switzerland. This article is an open access article distributed under the terms and conditions of the Creative Commons Attribution (CC BY) license (http://creativecommons.org/licenses/by/4.0/).
